# Long non-coding RNA XIST: a novel oncogene in multiple cancers

**DOI:** 10.1186/s10020-021-00421-0

**Published:** 2021-12-20

**Authors:** Jun Yang, Manlong Qi, Xiang Fei, Xia Wang, Kefeng Wang

**Affiliations:** 1 Department of Gastroenterology, Shengjing Hospital of China Medical University, Shenyang, 110004 China; 2 Department of Clinical Genetics, Shengjing Hospital of China Medical University, Shenyang, 110004 China; 3 Department of Urology, Shengjing Hospital of China Medical University, #36 Sanhao Street, Heping, Liaoning 110004 Shenyang, China

**Keywords:** Long non-coding RNA, XIST, Oncogene, Cancer

## Abstract

Long non-coding RNA (lncRNA) X-inactive specific transcript (XIST) is an important lncRNA derived from the XIST gene in mammals. XIST is abnormally expressed in numerous tumors, in most of which XIST functions as an oncogene. XIST is involved in multiple aspects of carcinogenesis, including tumor onset, progression, and prognosis. In our review, we collected and analyzed the recent studies on the impact of XIST in human tumor development. The multilevel molecular functions of XIST in human tumors are comprehensively reviewed to clarify the pathologic mechanisms and to offer a novel direction for further study.

## Introduction

Malignant tumors are the second leading cause of death worldwide and a threat to human health. Many studies have shown that approximately 70% of cardiovascular diseases can be cured by lifestyle adjustments. Therefore, tumors are likely to overtake cardiovascular disease as the leading cause of death within the next few years. Early detection, diagnosis, and treatment are important factors affecting the prognosis of tumors. Hence, researchers are attempting to identify the relevant novel biomarkers and prognostic factors.

Long non-coding RNAs (lncRNAs) are a cluster of RNA molecules between 200 and 100,000 nucleotides in length, and are involved in the regulation of many intracellular processes, with the exception of coding proteins (Spizzo et al. 2012). Recent studies have shown that lncRNAs are involved in X staining silencing, genomic imprinting, chromatin modification, transcriptional activation, transcriptional interference, nuclear transport, tumor regulation, and other important regulatory processes (Yue et al. 2014; Rafiee et al. 2018; Wang et al. 2017); however, the mechanism underlying lncRNA regulation of the onset and development of tumors has not been established.

LncRNA X-inactive specific transcript (XIST) was first reported by Brown et al. (1991) in 1991. XIST is a product of the XIST gene and a key regulatory factor of X-chromosome inactivation (XCI) in mammals. XCI is an epigenetic silencing of a random X chromosome in female cells to balance the level of X gene expression between males and females (Lyon 1972; Loda and Heard 2019). More recently, abnormal overexpression of XIST was identified in a variety of human malignant tumors, such as esophageal cancer (EC) (Wu et al. 2017a), gastric cancer (GC) (Chen et al. 2016), colorectal cancer (CRC) (Zhang et al. 2019a), pancreatic cancer (Sun et al. 2018a), hepatocellular carcinoma (HCC) (Liu and Xu 2019), laryngeal cancer (Xiao et al. 2019), lung cancer (Tantai et al. 2015), glioma (Yao et al. 2015), neuroblastoma (NB) (Zhang et al. 2019b), osteosarcoma (OS) (Li et al. 2017), bladder cancer (BC) (Xu et al. 2018a), retinoblastoma (RB) (Lyu et al. 2019), cervical cancer (CC) (Zhu et al. 2018a), thyroid cancer (Xu et al. 2018b), nasopharyngeal carcinoma (NPC) (Song et al. 2016), melanoma (Hao et al. 2021), and leukemia (Wang et al. 2020a). In our review, we have summarized the mechanism underlying XIST, as well as the clinical significance of XIST, in the occurrence and progression of tumors.

## XIST in various human tumors

XIST has been shown to be abnormally overexpressed in multiple cancers, exhibiting the properties of an oncogene in promoting tumor growth, invasion, metastasis, colony formation, and chemotherapy resistance (Fang et al. 2016; Zhang et al. 2017a; Li et al. 2019a). XIST also exhibits anti-tumor properties in a small subset of tumors, such as lymphomas (Parodi 2020). In addition, XIST displays an opposite effect in the same cancer, suggesting that XIST controls cancer development at multiple levels (Ma et al. 2020; Li et al. 2020a). In recent years, a number of studies have shown that XIST is involved in the clinicopathologic development of multiple cancers through post-transcriptional gene regulation. The specific mechanisms and functional characteristics of XIST in various human tumors are listed in Tables 1, 2, 3, 4, 5, 6, 7, 8.

**Table 1:** Functional characterization of XIST in digestive system tumors

Tumor types	Expression	Role	Function role	miRNAs	Related genes	References
Esophageal cancer	Upregulation	Oncogene	Proliferation, migration, and invasion	miR-101	EZH2	Wu et al. 2017a)
Esophageal cancer	Upregulation	Oncogene	Proliferation, apoptosis, migration, and invasion	miR-494	CDK6/JAK2/STAT3	Chen et al. 2019a)
Esophageal cancer	Upregulation	Oncogene	Cell cycle, proliferation, migration, invasion, and apoptosis	miR-129-5p	CCND1	Wang et al. 2021)
Gastric cancer	Upregulation	Oncogene	Proliferation, migration, and invasion	miR-101	EZH2	Chen et al. 2016)
Gastric cancer	Upregulation	Oncogene	Proliferation and invasion	miR-497	MACC1	Ma et al. 2017)
Gastric cancer	Upregulation	Oncogene	Proliferation, migration, and invasion	miR-185	TGF-β1	Zhang et al. 2018)
Gastric cancer	Upregulation	Oncogene	Proliferation, migration, and invasion	miR-337	JAK2	Zheng et al. 2020a)
Gastric cancer	Upregulation	Oncogene	Proliferation, migration, and apoptosis	miR-132	PXN	Li et al. 2020b)
Colorectal cancer	Upregulation	Oncogene	Proliferation	miR-132-3p	MAPK1	Song et al. 2017)
Colorectal cancer	Upregulation	Oncogene	Proliferation, migration, invasion, emt, and stem cell formation	miR-200b-3p	ZEB1	Chen et al. 2017)
Colorectal cancer	Upregulation	Oncogene	Migration and invasion	miR-137	EZH2	Liu et al. 2018)
Colorectal cancer	Upregulation	Oncogene	Proliferation and invasion	miR-34a	WNT1	Sun et al. 2018b)
Colorectal cancer	Upregulation	Oncogene	Growth, viability, apoptosis, and emt	miR-486b-5p	NRP-2	Liu et al. 2019)
Colorectal cancer	Upregulation	Oncogene	Migration, proliferation, emt, and apoptosis	miR-93-5p	HIF-1A	Yang et al. 2020)
Colorectal cancer	Upregulation	Oncogene	Proliferation, migration, invasion, and apoptosis	miR-338-3p	PAX5	Li et al. 2021)
Colorectal cancer	Upregulation	Oncogene	Proliferation, migration, invasion, and apoptosis	miR-497-5p	FOXK1	Wang et al. 2020b)
Colorectal cancer	Upregulation	Oncogene	Chemoresistance	miR-124	SGK1	Zhu et al. 2018b)
Colorectal cancer	Upregulation	Oncogene	Viability, proliferation, apoptosis, and chemoresistance	miR-30a-5p	ROR1	Zhang et al. 2019c)
Colorectal cancer	Upregulation	Oncogene	Proliferation, migration, invasion, and chemoresistance	miR-137	PKM2/PKM1	Zheng et al. 2020)
Pancreatic cancer	Upregulation	Oncogene	Proliferation, migration, and invasion	miR-34a-5p	/	Sun et al. 2018a)
Pancreatic cancer	Upregulation	Oncogene	Proliferation	miR-133a	EGFR	Wei et al. 2017)
Pancreatic cancer	Upregulation	Oncogene	Proliferation	miR-140/124	iASPP	Liang et al. 2017)
Pancreatic cancer	Upregulation	Oncogene	Emt	miR-34a	YAP	Zou et al. 2020)
Pancreatic cancer	Upregulation	Oncogene	Migration, invasion, and emt	miR-429	ZEB1	Shen et al. 2019)
Pancreatic cancer	Upregulation	Oncogene	Proliferation, migration, and invasion	miR-141-3p	TGF-β2	Sun and Zhang 2019)
Pancreatic cancer	Upregulation	Oncogene	Proliferation	miR-137	Notch1	Liu et al. 2020)
Hepatocellular carcinoma	Upregulation	Oncogene	Proliferation	miR-200b-3p	/	Liu and Xu 2019)
Hepatocellular carcinoma	Upregulation	Oncogene	Proliferation and apoptosis	miR-488	/	Dong et al. 2020)
Hepatocellular carcinoma	Upregulation	Oncogene	Proliferation and apoptosis	miR-139-5p	PDK1	Mo et al. 2017)
Hepatocellular carcinoma	Upregulation	Oncogene	Proliferation, migration, and invasion	miR-194-5p	MAPK1	Kong et al. 2018)

**Table 2:** Main characteristics of the studies included in the review of digestive system tumors

Study	Tumor types	Sample size (Normal: Tumor)	Detection Method	P value	TNM (p value)	LNM (p value)	DM (p value)	OS (p value)	References
Wu	Esophageal cancer	(127: 127)	qRT-PCR	p = 0.0092	p = 0.000	/	/	p = 0.005	Wu et al. 2017a)
Chen	Esophageal cancer	(78: 78)	qRT-PCR	p < 0.001	/	/	/	/	Chen et al. 2019a)
Wang	Esophageal cancer	(42: 42)	qRT-PCR	p < 0.001	p = 0.0064	/	/	p = 0.0039	Wang et al. 2021)
Chen	Gastric cancer	(106: 106)	qRT-PCR	p < 0.001	/	/	p = 0.033	p = 0.002	Chen et al. 2016)
Ma	Gastric cancer	(98: 98)	qRT-PCR	p < 0.05	p = 0.005	p = 0.002	/	p < 0.05	Ma et al. 2017)
Li	Gastric cancer	(65: 65)	qRT-PCR	p < 0.05	/	/	/	/	Li et al. 2020b)
Li	Gastric cancer	(98: 98)	qRT-PCR	p < 0.05	p = 0.0077	p = 0.014	/	/	Li et al. 2020)
Zhang	Colorectal cancer	(196: 196)	qRT-PCR	p < 0.001	/	/	p < 0.001	p < 0.001	Zhang et al. 2019a)
Yu	Colorectal cancer	(41: 94)	qRT-PCR	p < 0.05	/	/	/	p < 0.001	Yu et al. 2020)
Yang	Colorectal cancer	(37: 37)	qRT-PCR	p < 0.0001	/	/	p = 0.008	/	Yang et al. 2020)
Song	Colorectal cancer	(50: 50)	qRT-PCR	p < 0.05	p = 0.034	/	/	/	Song et al. 2017)
Chen	Colorectal cancer	(115: 115)	qRT-PCR	p < 0.05	/	/	/	p = 0.01	Chen et al. 2017)
Liu	Colorectal cancer	(20: 20)	qRT-PCR	p < 0.001	/	/	/	/	Liu et al. 2018)
Sun	Colorectal cancer	(120: 120)	qRT-PCR	p < 0.01	p = 0.005	p = 0.035	p = 0.02	p < 0.05	Sun et al. 2018b)
Liu	Colorectal cancer	(317: 317)	qRT-PCR	p < 0.05	p = 0.04	p < 0.001	/	p < 0.001	Liu et al. 2019)
Yang	Colorectal cancer	(36: 36)	qRT-PCR	p < 0.001	p = 0.0333	/	/	/	Yang et al. 2020)
Li	Colorectal cancer	(30: 30)	qRT-PCR	p < 0.05	p = 0.028	/	/	/	Li et al. 2021)
Wang	Colorectal cancer	(54: 54)	qRT-PCR	p < 0.01	/	/	/	/	Wang et al. 2020b)
Zhang	Colorectal cancer	(294: 294)	qRT-PCR	p < 0.05	/	p = 0.037	/	p < 0.001	Zhang et al. 2019c)
Sun	Pancreatic cancer	(139: 139)	qRT-PCR	p < 0.001	/	/	/	p < 0.001	Sun et al. 2018a)
Wei	Pancreatic cancer	(64: 64)	qRT-PCR	p < 0.01	p = 0.023	/	/	p = 0.002	Wei et al. 2017)
Liang	Pancreatic cancer	(73: 73)	qRT-PCR	p < 0.01	/	/	/	p = 0.003	Liang et al. 2017)
Shen	Pancreatic cancer	(120: 120)	qRT-PCR	p < 0.001	/	/	/	/	Shen et al. 2019)
Sun	Pancreatic cancer	(30: 30)	qRT-PCR	p < 0.01	/	/	/	/	Sun and Zhang 2019)
Liu	Pancreatic cancer	(40: 40)	qRT-PCR	p < 0.001	/	/	/	/	Liu et al. 2020)
Liu	Hepatocellular carcinoma	(55: 55)	qRT-PCR	p < 0.05	/	/	/	/	Liu and Xu 2019)
Dong	Hepatocellular carcinoma	(69: 69)	qRT-PCR	p < 0.001	/	/	/	/	Dong et al. 2020)
Mo	Hepatocellular carcinoma	(88: 88)	qRT-PCR	p < 0.05	/	/	/	/	Mo et al. 2017)
Kong	Hepatocellular carcinoma	(52: 52)	qRT-PCR	p < 0.05	/	/	/	p < 0.05	Kong et al. 2018)

**Table 3:** Functional characterization of XIST in respiratory system tumors

Tumor types	Expression	Role	Function role	miRNAs	Related genes	References
Laryngeal cancer	Upregulation	Oncogene	Proliferation, migration, and invasion	miR-124	EZH2	Xiao et al. 2019)
Laryngeal cancer	Upregulation	Oncogene	Proliferation, migration, invasion, and apoptosis	miR-144	IRS1	Cui et al. 2020)
Laryngeal cancer	Upregulation	Oncogene	Proliferation, migration, invasion, and apoptosis	miR-125b-5p	TRIB2	Liu et al. 2020)
Lung cancer	Upregulation	Oncogene	Proliferation, invasion, and apoptosis	miR-186-5p	/	Wang et al. 2017b)
Lung cancer	Upregulation	Oncogene	Proliferation, migration, invasion, and apoptosis	miR-449a	BCL2	Zhang et al. 2017b)
Lung cancer	Upregulation	Oncogene	Proliferation and apoptosis	miR-140	iASPP	Tang et al. 2017)
Lung cancer	Upregulation	Oncogene	Growth and motility	miR-374a	LARP1	Xu et al. 2017)
Lung cancer	Upregulation	Oncogene	Proliferation and emt	miR-137	Notch1	Wang et al. 2018)
Lung cancer	Upregulation	Oncogene	Proliferation and invasion	miR-744	RING1	Wang et al. 2019)
Lung cancer	Upregulation	Oncogene	Proliferation and apoptosis	miR-335	SOD2	Liu et al. 2019)
Lung cancer	Upregulation	Oncogene	Proliferation, migration, and cell cycle	miR-16	CDK8	Zhou et al. 2019)
Lung cancer	Upregulation	Oncogene	Proliferation, migration, invasion, and emt	miR-212-3p	CBLL1	Qiu et al. 2019)
Lung cancer	Upregulation	Oncogene	Proliferation, migration, invasion, and apoptosis	miR-363-3p	MDM2	Rong et al. 2020)
Lung cancer	Upregulation	Oncogene	Proliferation, migration, invasion, and apoptosis	miR-142-5p	PAX6	Jiang et al. 2020)
Lung cancer	Upregulation	Oncogene	Chemoresistance	let-7i	BAG-1	Sun et al. 2017)
Lung cancer	Upregulation	Oncogene	Autophagy and chemoresistance	miR-17	ATG7	Sun et al. 2017)
Lung cancer	Upregulation	Oncogene	Proliferation, migration, apoptosis, and chemoresistance	miR-144-3p	MDR1	Tian et al. 2019)

**Table 4:** Main characteristics of the studies included in the review of respiratory system tumors

Study	Tumor types	Sample size (Normal: Tumor)	Detection Method	P value	TNM (p value)	LNM (p value)	DM (p value)	OS (p value)	References
Cui	Laryngeal cancer	(48: 48)	qRT-PCR	p < 0.01	/	/	/	/	Cui et al. 2020)
Liu	Laryngeal cancer	(40: 40)	qRT-PCR	p < 0.05	p = 0.005	/	p = 0.011	p = 0.0423	Liu et al. 2020)
Wang	Lung cancer	(30: 30)	qRT-PCR	p < 0.001	/	/	/	/	Wang et al. 2017b)
Wang	Lung cancer	(33: 33)	qRT-PCR	p < 0.05	/	/	/	/	Wang et al. 2018)
Wang	Lung cancer	(20: 20)	qRT-PCR	p < 0.001	p = 0.000	/	/	p = 0.0264	Wang et al. 2019)
Liu	Lung cancer	(45: 45)	qRT-PCR	p < 0.01	/	/	/	/	Liu et al. 2019)
Zhou	Lung cancer	(15: 15)	qRT-PCR	p < 0.05	/	/	/	/	Zhou et al. 2019)
Qiu	Lung cancer	(33: 33)	qRT-PCR	p < 0.05	/	/	/	p < 0.05	Qiu et al. 2019)
Rong	Lung cancer	(35: 35)	qRT-PCR	p < 0.05	/	/	/	/	Rong et al. 2020)
Jiang	Lung cancer	(30: 30)	qRT-PCR	p < 0.05	/	/	/	/	Jiang et al. 2020)
Sun	Lung cancer	(50: 50)	qRT-PCR	p < 0.05	p = 0.045	/	/	/	Sun et al. 2017)
Xu	Lung cancer	(30: 30)	qRT-PCR	p < 0.05	/	/	/	/	Xu et al. 2020)

**Table 5:** Functional characterization of XIST in nervous system tumors

Tumor types	Expression	Role	Function role	miRNAs	Related genes	References
Glioma	Upregulation	Oncogene	Proliferation, migration, invasion, and apoptosis	miR-152	/	Yao et al. 2015)
Glioma	Upregulation	Oncogene	Tumorigenicity and angiogenesis	miR-429	/	Cheng et al. 2017)
Glioma	Upregulation	Oncogene	Angiogenesis	miR-137	ZO-2/FOXC1	Yu et al. 2017)
Glioma	Upregulation	Oncogene	Proliferation	miR-137	Rac1	Wang et al. 2017c)
Glioma	Upregulation	Oncogene	Viability, migration, invasion, apoptosis, and glucose metabolism	miR-126	IRS1/PI3K/Akt	Cheng et al. 2020)
Glioma	Upregulation	Oncogene	Proliferation, metastasis, and emt	miR-133a	SOX4	Luo et al. 2020)
Glioma	Upregulation	Oncogene	Proliferation, migration, invasion, and apoptosis	miR-204-5p	Bcl-2	Shen et al. 2020)
Glioma	Upregulation	Oncogene	Stemness	miR-152	KLF4	Gong et al. 2021)
Glioma	Upregulation	Oncogene	Proliferation, invasion, apoptosis, and radio-sensitivity	miR-329-3p	CREB1	Wang et al. 2020c)
Neuroblastoma	Upregulation	Oncogene	Cell cycle, proliferation, and radio-sensitivity	miR-375	L1CAM	Yang et al. 2020)

**Table 6:** Main characteristics of the studies included in the review of nervous system tumors

Study	Tumor types	Sample size (Normal: Tumor)	Detection Method	P value	TNM (p value)	LNM (p value)	DM (p value)	OS (p value)	References
Wang	Glioma	(18: 30)	qRT-PCR	p < 0.05	p = 0.014	/	/	/	Wang et al. 2017c)
Du	Glioma	(69: 69)	qRT-PCR	p < 0.01	p = 0.079	/	/	p = 0.0007	Du et al. 2017)
Wang	Glioma	(30: 30)	qRT-PCR	p < 0.05	/	/	/	/	Wang et al. 2020c)
Zhang	Neuroblastoma	(30: 30)	qRT-PCR	p < 0.05	p = 0.011	/	/	/	Zhang et al. 2019b)
Yang	Neuroblastoma	(20: 36)	qRT-PCR	p < 0.05	/	/	/	/	Yang et al. 2020)

**Table 7:** Functional characterization of XIST in other system tumors

Tumor types	Expression	Role	Function role	miRNAs	Related genes	References
Osteosarcoma	Upregulation	Oncogene	Proliferation and invasion	miR-137	/	Li et al. 2019b)
Osteosarcoma	Upregulation	Oncogene	Proliferation and invasion	miR-320b	RAP2B	Lv et al. 2018)
Osteosarcoma	Upregulation	Oncogene	Proliferation and invasion	miR-193a-3p	RSF1	Wu et al. 2017b)
Osteosarcoma	Upregulation	Oncogene	Proliferation, autophagy, and apoptosis	miR-375-3p	AKT/mTOR	Sun et al. 2019)
Osteosarcoma	Upregulation	Oncogene	Migration, invasion, and emt	miR-153	SNAIL	Wen et al. 2020)
Osteosarcoma	Upregulation	Oncogene	Proliferation and apoptosis	miR-124-3p	iASPP	Hai et al. 2021)
Bladder cancer	Upregulation	Oncogene	Clone formation and emt	miR-200c	/	Xu et al. 2018a)
Bladder cancer	Upregulation	Oncogene	Proliferation and migration	miR-133a	/	Zhou et al. 2019)
Bladder cancer	Upregulation	Oncogene	Proliferation, migration, and invasion	miR-124	AR	Xiong et al. 2017)
Bladder cancer	Upregulation	Oncogene	Proliferation, migration, invasion, and apoptosis	miR-139-5p	Wnt/β-catenin	Hu et al. 2017)
Bladder cancer	Upregulation	Oncogene	Proliferation, migration, invasion, and emt	miR-335	/	Chen et al. 2020)
Retinoblastoma	Upregulation	Oncogene	Proliferation, invasion, apoptosis, and emt	miR-142-5p	/	Xu and Tian 2020)
Retinoblastoma	Upregulation	Oncogene	Proliferation, cell cycle, and apoptosis	miR-124	STAT3	Hu et al. 2018)
Retinoblastoma	Upregulation	Oncogene	Proliferation, migration, invasion, apoptosis, and emt	miR-101	ZEB1/ZEB2	Cheng et al. 2019)
Retinoblastoma	Upregulation	Oncogene	Proliferation and invasion	miR-140-5p	SOX4	Wang et al. 2020d)
Retinoblastoma	Upregulation	Oncogene	Proliferation, invasion, apoptosis, and emt	miR-200a-3p	NRP1	Zhao et al. 2020)
Retinoblastoma	Upregulation	Oncogene	Proliferation, migration, invasion, autophagy, and apoptosis	miR-361-3p	STX17	Yang et al. 2020)
Retinoblastoma	Upregulation	Oncogene	Proliferation, autophagy, and drug sensitivity	miR-204-5p	/	Yao et al. 2020)
Cervical cancer	Upregulation	Oncogene	Proliferation, apoptosis, invasion, and emt	miR-200a	Fus	Zhu et al. 2018a)
Cervical cancer	Upregulation	Oncogene	Proliferation and apoptosis	miR-140-5p	ORC1	Chen et al. 2019b)
Cervical cancer	Upregulation	Oncogene	Proliferation, migration, invasion, and apoptosis	miR-889-3p	SIX1	Liu et al. 2020)
Thyroid cancer	Upregulation	Oncogene	Proliferation, migration, and invasion	miR-141	/	Xu et al. 2018b)
Thyroid cancer	Upregulation	Oncogene	Proliferation	miR-34a	MET-PI3K-AKT	Liu et al. 2018)
Thyroid cancer	Upregulation	Oncogene	Migration and invasion	miR-101-3p	CLDN1	Du et al. 2021)
Nasopharyngeal cancer	Upregulation	Oncogene	Growth	miR-34a-5p	E2F3	Song et al. 2016)
Nasopharyngeal cancer	Upregulation	Oncogene	Proliferation and radiosensitivity	miR-29c	/	Han et al. 2017)
Nasopharyngeal cancer	Upregulation	Oncogene	Proliferation, invasion, and apoptosis	miR-491-5p	/	Cheng et al. 2018)
Nasopharyngeal cancer	Upregulation	Oncogene	Proliferation, metastasis, and emt	miR-148a-3p	ADAM17	Shi et al. 2020)
Nasopharyngeal cancer	Upregulation	Oncogene	Glycolysis, migration, and invasion	miR-381-3p	NEK5	Zhao et al. 2020)
Melanoma	Upregulation	Oncogene	Proliferation and apoptosis	miR-23a-3p	GINS2	Hao et al. 2021)
Leukemia	Upregulation	Oncogene	Drug resistance, viability, and apoptosis	miR-29a	MYC	Wang et al. 2020a)

**Table 8:** Main characteristics of the studies included in the review of other system tumors

Study	Tumor types	Sample size (Normal: Tumor)	Detection Method	P value	TNM (p value)	LNM (p value)	DM (p value)	OS (p value)	References
Li	Osteosarcoma	(145: 145)	qRT-PCR	p < 0.01	p = 0.007	/	p = 0.008	p < 0.001	Li et al. 2017)
Xu	Osteosarcoma	(66: 66)	qRT-PCR	p < 0.01	/	/	/	/	Xu et al. 2017)
Wang	Osteosarcoma	(64: 64)	qRT-PCR	p < 0.01	/	/	p = 0.012	p = 0.034	Wang et al. 2019)
Li	Osteosarcoma	(35: 35)	qRT-PCR	p < 0.01	/	/	/	/	Li et al. 2019b)
Lv	Osteosarcoma	(34: 34)	qRT-PCR	p < 0.05	/	/	/	/	Lv et al. 2018)
Wen	Osteosarcoma	(30: 30)	qRT-PCR	p < 0.001	/	/	/	/	Wen et al. 2020)
Hai	Osteosarcoma	(15: 38)	qRT-PCR	p < 0.001	/	/	/	p = 0.0221	Hai et al. 2021)
Zhou	Bladder cancer	(52: 52)	qRT-PCR	p < 0.01	p = 0.001	p = 0.001	p = 0.001	p = 0.039	Zhou et al. 2019)
Xiong	Bladder cancer	(67: 67)	qRT-PCR	p < 0.01	p = 0.01	/	/	/	Xiong et al. 2017)
Hu	Bladder cancer	(52: 52)	qRT-PCR	p < 0.05	p = 0.012	p = 0.042	/	p = 0.0332	Hu et al. 2017)
Xu	Retinoblastoma	(53: 87)	qRT-PCR	p < 0.05	/	/	/	p < 0.05	Xu and Tian 2020)
Hu	Retinoblastoma	(6: 30)	qRT-PCR	p < 0.01	p < 0.01	/	/	/	Hu et al. 2018)
Cheng	Retinoblastoma	(7: 53)	qRT-PCR	p < 0.05	/	/	/	/	Cheng et al. 2019)
Wang	Retinoblastoma	(8: 20)	qRT-PCR	p < 0.001	/	/	/	/	Wang et al. 2020d)
Zhao	Retinoblastoma	(54: 54)	qRT-PCR	p < 0.001	/	/	/	/	Zhao et al. 2020)
Yang	Retinoblastoma	(30: 30)	qRT-PCR	p < 0.05	/	/	/	/	Yang et al. 2020)
Yao	Retinoblastoma	(6: 25)	qRT-PCR	p < 0.05	/	/	/	/	Yao et al. 2020)
Zhu	Cervical cancer	(52: 52)	qRT-PCR	p < 0.01	p = 0.04	/	p = 0.027	p = 0.015	Zhu et al. 2018a)
Chen	Cervical cancer	(30: 30)	qRT-PCR	p < 0.001	p = 0.033	p = 0.038	/	/	Chen et al. 2019b)
Liu	Cervical cancer	(35: 35)	qRT-PCR	p < 0.05	p < 0.05	/	/	p < 0.01	Liu et al. 2020)
Xu	Thyroid cancer	(36: 36)	qRT-PCR	p < 0.01	p < 0.01	p < 0.01	/	/	Xu et al. 2018b)
Liu	Thyroid cancer	(77: 77)	qRT-PCR	p < 0.01	/	/	/	p = 0.025	Liu et al. 2018)
Du	Thyroid cancer	(24: 24)	qRT-PCR	p < 0.01	/	/	/	/	Du et al. 2021)
Song	Nasopharyngeal cancer	(108: 108)	qRT-PCR	p < 0.05	/	/	/	p = 0.0005	Song et al. 2016)
Cheng	Nasopharyngeal cancer	(10: 20)	qRT-PCR	p < 0.05	/	/	/	/	Cheng et al. 2018)
Shi	Nasopharyngeal cancer	(40: 40)	qRT-PCR	p < 0.05	/	/	/	/	Shi et al. 2020)
Zhao	Nasopharyngeal cancer	(25: 25)	qRT-PCR	p < 0.05	/	/	/	/	Zhao et al. 2020)
Hao	Melanoma	(15: 15)	qRT-PCR	p < 0.01	/	/	/	/	Hao et al. 2021)
Wang	Leukemia	(20: 62)	qRT-PCR	p < 0.05	/	/	/	/	Wang et al. 2020a)

### The role of XIST in digestive system tumors

#### XIST in esophageal cancer (EC)

EC ranked 7th in morbidity (604,100 new cases) and mortality (544,076 deaths) worldwide in 2020, with 70% of cases occurring in men (Sung et al. 2021). Esophageal squamous cell carcinoma (ESCC) is the histological type most common in low income Asian and African countries. The main causes of ESCC may be nutritional deficiencies, nitrosamine use, betel nut chewing, and consumption of preserved vegetables (Sung et al. 2021). Esophageal adenocarcinoma is most common in Western high income countries and is mainly caused by alcohol abuse, cigarette smoking, overweight, gastroesophageal reflux disease, and Barrett's esophagus (Sung et al. 2021). Therefore, further study of the molecular mechanism underlying EC is warranted.

**Fig. 1:**
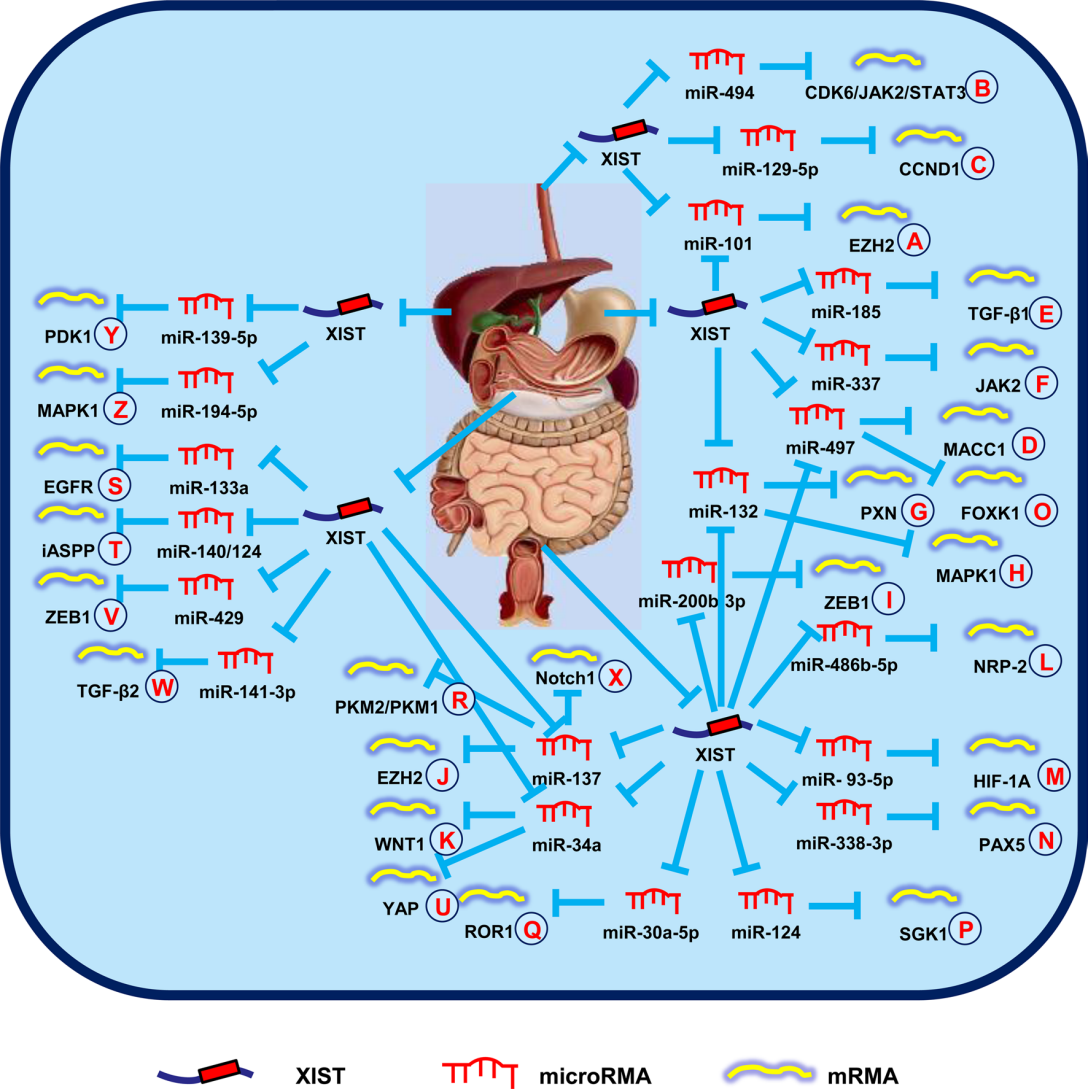
XIST mediates mechanisms involved in digestive system tumors. **A** XIST could promote the expression of EZH2 by targeting miR-101. **B** XIST could promote the expression of CDK6/JAK2/STAT3 by targeting miR-494. **C** XIST could promote the expression of CCND1 by targeting miR-129-5p. **D** XIST could promote the expression of MACC1 by targeting miR-497. **E** XIST could promote the expression of TGF-β1 by targeting miR-185. **F** XIST could promote the expression of JAK2 by targeting miR-337. **G** XIST could promote the expression of PXN by targeting miR-132. **H** XIST could promote the expression of MAPK1 by targeting miR-132-3p. **I** XIST could promote the expression of ZEB1 by targeting miR-200b-3p. **J** XIST could promote the expression of EZH2 by targeting miR-137. **K** XIST could promote the expression of WNT1 by targeting miR-34a. **L** XIST could promote the expression of NRP-2 by targeting miR-486b-5p. **M** XIST could promote the expression of HIF-1A by targeting miR-93-5p. **N** XIST could promote the expression of PAX5 by targeting miR-338-3p. **O** XIST could promote the expression of FOXK1 by targeting miR-497-5p. **P** XIST could promote the expression of SGK1 by targeting miR-124. **Q** XIST could promote the expression of ROR1 by targeting miR-30a-5p. **R** XIST could promote the expression of PKM2/PKM1 by targeting miR-137. **S** XIST could promote the expression of EGFR by targeting miR-133a. **T** XIST could promote the expression of iASPP by targeting miR-140/124. **U** XIST could promote the expression of YAP by targeting miR-34a. **V** XIST could promote the expression of ZEB1 by targeting miR-429. **W** XIST could promote the expression of TGF-β2 by targeting miR-141-3p. **X** XIST could promote the expression of Notch1 by targeting miR-137. **Y** XIST could promote the expression of PDK1 by targeting miR-139-5p. **Z** XIST could promote the expression of MAPK1 by targeting miR-194-5p

Wu et al. (2017a) reported that XIST is upregulated in ESCCs and performed an oncogenic progression through regulation of the miR-101/EZH2 signal pathway (Fig. 1A). Then, another group reported that XIST induced carcinogenesis through the miR-494/CDK6/JAK2/STAT3 signal pathway in EC, which provided a potential means for investigation of EC (Fig. 1B) (Chen et al. 2019a). Additionally, Wang et al. (2021) showed that downregulation of XIST suppressed the malignant behaviors in part by antagonizing the miR-129-5p/CCND1 signal pathway in EC (Fig. 1C).

Thus, XIST provides a molecular mechanism for inhibiting EC progression.

#### XIST in gastric cancer (GC)

It is estimated that GC caused 1,089,103 new cases and 768,793 deaths in 2020, ranking 6th in incidence and 4th in mortality globally (Sung et al. 2021). The main causes of GC are *Helicobacter pylori* infection, alcohol consumption, low fruit intake, tobacco smoking, and high consumption of processed meat (Sung et al. 2021). Thus, it is important to search for diagnostic and therapeutic strategies to improve GC patient outcomes.

Chen et al. ( 2016) reported that XIST was responsible for the aggressive phenotype of GC and was involved in post-transcriptional control via the miR-101/EZH2 signal pathway (Fig. 1A). Subsequently, Ma et al. (2017) reported that XIST promoted cell growth and invasion via miR-497/MACC1 signals, which suggested a potential prognostic factor for GC (Fig. 1D). Another research group demonstrated that XIST functioned as a competing endogenous RNA (ceRNA) to modulate TGF-β1 expression by sequestering miR-185 in GC (Fig. 1E) (Zhang et al. 2018). In addition, Zheng et al. (2020a) showed that XIST silencing inhibited cell growth and migration via regulation of the miR-337/JAK2 signal pathway in GC (Fig. 1F). Li et al. (2020b) showed that XIST increased PXN expression through combination with miR-132 (Fig. 1G). Knockdown of XIST reversed the anti-tumor effect exerted by PXN, which highlighted the therapeutic role in GC. Six months later, Li et al. (2020) suggested that XIST promoted the progression of GC via upregulation of HNF4A enrichment in the promoter region of EPHA1.

Taken together, the above data indicated that XIST was responsible for an oncogene in GC development.

#### XIST in colorectal cancer (CRC)

It has been estimated that there will be 1,880,725 new cases of CRC and 915,880 deaths in 2020, accounting for approximately one in 10 new cases and deaths (Sung et al. 2021). CRC ranks third in morbidity, but second in mortality (Sung et al. 2021). Reducing physical activity, gaining excess weight, drinking too much alcohol, smoking cigarettes, and eating red or processed meat are risk factors for CRC, while calcium supplementation and adequate intake of whole grains, fiber, and dairy products appear to reduce the risk (Siegel et al. 2020). Therefore, the pathogenesis underlying CRC is essential for identifying the therapeutic targets.

Zhang et al. (2019a) analyzed the correlation between XIST expression and the clinicopathological features of CRC. It has been reported that XIST was elevated in CRC and was used as an independent risk factor for the prognosis of CRC. Similar results indicated that patients with high XIST expression had worse survival rates, higher lymphatic metastases, shorter life cycles, and lower differentiation than patients with low XIST expression. Therefore, serum XIST expression may contribute to the diagnosis and prognosis of CRC (Yu et al. 2020). Another study concluded that METTLE14 downregulated and increased XIST expression by m6A-YTHDF2 to promote cell growth and invasion in CRC (Yang et al. 2020).

The study conducted by Song et al. (2017) indicated that XIST promoted cell growth by suppressing the miR-132-3p/MAPK1 signal pathway and was therefore a potential oncogenic target of CRC (Fig. 1H). Subsequently, the role of lncRNA-miRNA-mRNA axis in CRC became the focus of research by a number of groups. Specifically, it was shown that XIST promoted CRC cell malignant activities and upregulated ZEB1 (Fig. 1I), EZH2 (Fig. 1J), WNT1 (Fig. 1K), NRP-2 (Fig. 1L), HIF-1A (Fig. 1M), PAX5 (Fig. 1N), and FOXK1 (Fig. 1O) by modulating miR-200b-3p (Chen et al. 2017), miR-137 (Liu et al. 2018), miR-34a (Sun et al. 2018b), miR-486-5p (Liu et al. 2019), miR-93-5p (Yang et al. 2020), miR-338-3p (Li et al. 2021), and miR-497-5p (Wang et al. 2020b), respectively.

In addition, XIST affects chemotherapy resistance of CRC. Xiao et al. (2017) showed that XIST was involved in 5-fluorouracil (5FU) resistance by promoting thymidylate synthase expression, thus XIST silencing is a potentially new therapeutic strategy to defeat 5FU resistance in patients with CRC. Corollary studies showed that XIST played a regulatory role in doxorubicin resistance via the miR-124/SGK1 signal pathway, which provided a novel way to thwart chemotherapy resistance in CRC (Fig. 1P) (Zhu et al. 2018b). Similarly, Zhang et al. (2019c) concluded that XIST influenced chemosensitivity via the miR-30a-5p/ROR1 axis and atractylenolide II enhanced the chemotherapeutic sensitivity of CRC cells (Fig. 1Q). Moreover, Zheng et al. (2020) demonstrated that XIST/miR-137 signals enhanced glycolysis and chemotherapy tolerance of CRC by increasing the PKM2-to-PKM1 ratio, thus providing another option for improving the efficacy of chemotherapy in CRC patients (Fig. 1R).

Overall, these data supported the essential role of XIST in CRC carcinogenesis, representing novel diagnostic and therapeutic targets.

#### XIST in pancreatic cancer

Due to the poor prognosis, pancreatic cancer has nearly as many deaths (466,003) as new cases (495,773), making pancreatic cancer the 7th leading cause of death for both men and women (Sung et al. 2021). In many countries, morbidity and mortality rates have remained stable or increased slightly, reflecting the growing prevalence of diabetes, obesity, and alcohol consumption. Therefore, further investigation of the pathogenic mechanism underlying pancreatic cancer is needed.

Sun et al. (2018a) reported that XIST was increased in pancreatic cancer tissues and upregulation of XIST promoted cell growth, migration, and invasion of pancreatic cancer. A mechanistic analysis showed that miR-34a-5p was a target of XIST and could rescue the malignant activities mediated by XIST in pancreatic cancer. As a result, a number of investigators focused on the role of XIST in the regulation of pancreatic cancer. It was further demonstrated that XIST promoted pancreatic cancer cell progression through upregulating EGFR (Fig. 1S), iASSP (Fig. 1T), YAP (Fig. 1U), ZEB1 (Fig. 1V), TGF-β2 (Fig. 1W), and Notch1 (Fig. 1X) by modulating miR-133a (Wei et al. 2017), miR-140/miR-124 (Liang et al. 2017), miR-34a (Zou et al. 2020), miR-429 (Shen et al. 2019), miR-141-3p (Sun and Zhang 2019), and miR-137 (Liu et al. 2020), respectively.

These findings convincingly indicated that XIST provided a diagnostic and therapeutic option for pancreatic cancer.

#### XIST in hepatocellular carcinoma (HCC)

Primary liver cancer is the 7th most common cancer and the 3rd cause of deaths globally, with approximately 905,677 new cases and 830,180 deaths (Sung et al. 2021). HCC accounts for 75–85% of primary liver cancers, the main risk factors of which are chronic hepatitis B or C virus infection, aflatoxin-contaminated food, excess body weight, heavy alcohol consumption, cigarette smoking, and type 2 diabetes (Sung et al. 2021). Therefore, it is important to identify the diagnostic biomarker and therapeutic target of HCC.

Liu et al. (2019) showed that XIST enhanced cell proliferation by targeting miR-200b-3p, which implied that XIST was a novel therapeutic target in HCC. Another study showed that suppression of XIST inhibited cell growth and promoted cell apoptosis by regulating miR-488 expression in HCC (Dong et al. 2020). Recently, the mechanism underlying XIST regulation of the progression of HCC has been shown to involve upregulation of PDK1 (Fig. 1Y) and MAPK1 (Fig. 1Z) by modulating miR-139-5p (Mo et al. 2017) and miR-194-5p (Kong et al. 2018), respectively.

These data demonstrated that XIST functioned as a novel prognostic marker and therapeutic target for HCC.

### The role of XIST in respiratory system tumors

#### XIST in laryngeal cancer

Laryngeal cancer belongs to head and neck tumors based on anatomic site and respiratory system tumors based on function. Laryngeal squamous cell carcinoma (LSCC) is the most common pathologic type of laryngeal cancer. It has been estimated that there will be 12,620 new cases of LSCC and 3770 deaths in the United States in 2021 (Siegel et al. 2021). Therefore, it is important to clarify the pathogenesis underlying laryngeal cancer to develop optimal treatment regimens.

**Fig. 2:**
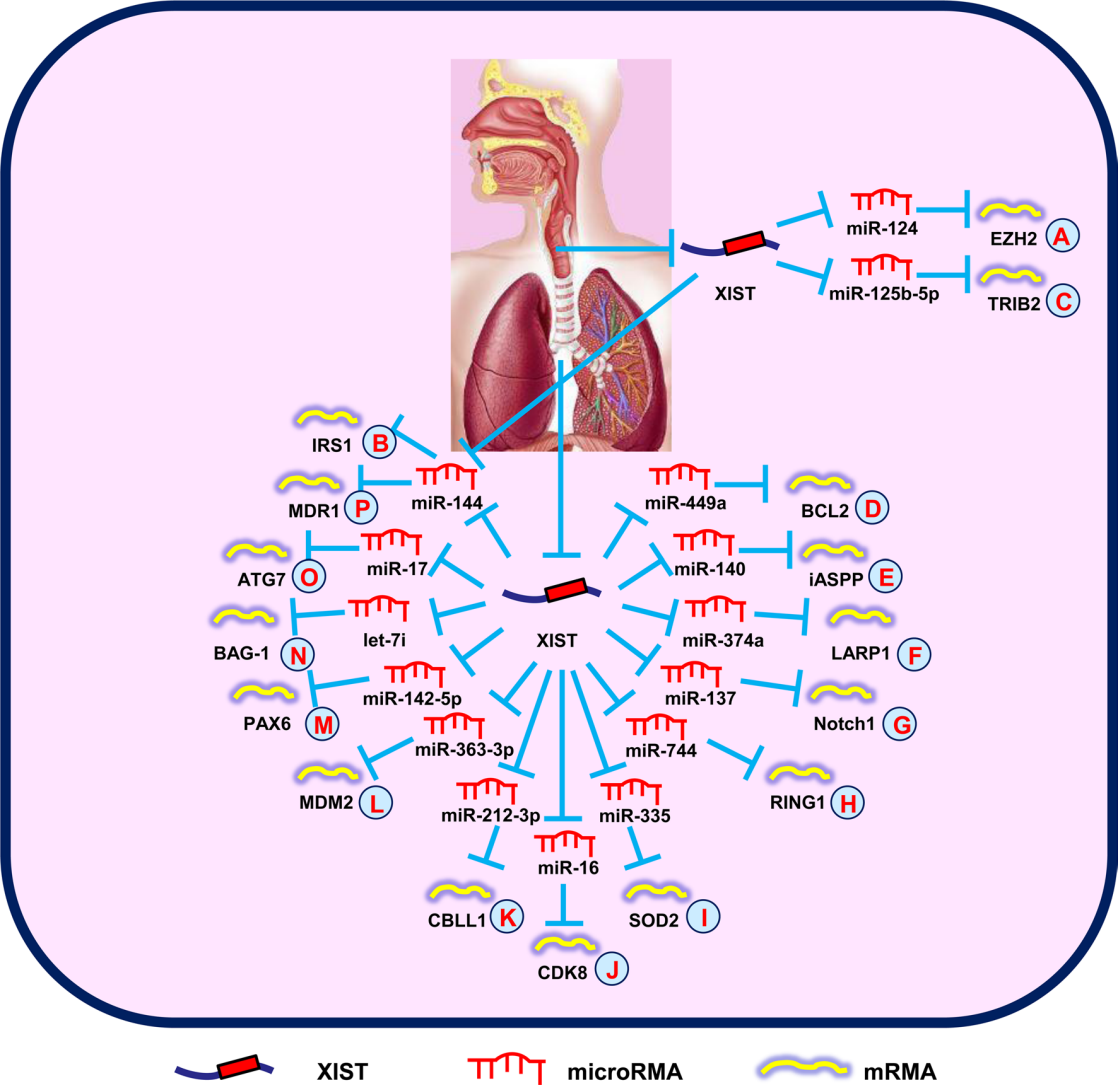
XIST mediates mechanisms involved in respiratory system tumors. **A** XIST could promote the expression of EZH2 by targeting miR-124. **B** XIST could promote the expression of IRS1 by targeting miR-144. **C** XIST could promote the expression of TRIB2 by targeting miR-125b-5p. **D** XIST could promote the expression of BCL2 by targeting miR-449a. **E** XIST could promote the expression of iASPP by targeting miR-140. **F** XIST could promote the expression of LARP1 by targeting miR-374a. **G** XIST could promote the expression of Notch1 by targeting miR-137. **H** XIST could promote the expression of RING1 by targeting miR-744. **I** XIST could promote the expression of SOD2 by targeting miR-335. **J** XIST could promote the expression of CDK8 by targeting miR-16. **K** XIST could promote the expression of CBLL1 by targeting miR-212-3p. **L** XIST could promote the expression of MDM2 by targeting miR-363-3p. **M** XIST could promote the expression of PAX6 by targeting miR-142-5p. **N** XIST could promote the expression of BAG-1 by targeting let-7i. **O** XIST could promote the expression of ATG7 by targeting miR-17. **P** XIST could promote the expression of MDR1 by targeting miR-144-3p

Xiao et al. (2019) showed that XIST expression was highly increased in LSCC. XIST has an important role in the proliferation, invasion, and metastasis of LSCC by regulating the miR-124/EZH2 signals (Fig. 2A). Similar results indicated that XIST promoted carcinogenic cell behavior by regulating the miR-144/IRS1 axis in LSCC, suggesting a potential therapeutic target for LSCC patient treatment (Fig. 2B) (Cui et al. 2020). Furthermore, Liu et al. (2020) reported that XIST upregulated TRIB2 expression in part by sequestration of miR-125b-5p, which in turn expedited LSCC cell progression (Fig. 2C).

In summary, XIST has a key role in the diagnosis and prognosis of LSCC.

#### XIST in lung cancer

With an estimated 2,206,771 new cases and 1,796,144 deaths annually, lung cancer ranks 2nd in morbidity and 1st in mortality (Sung et al. 2021). Approximately two-thirds of lung cancer deaths worldwide can be attributed to smoking (Sung et al. 2021). Other factors include occupational exposure and environmental pollution (Turner et al. 2020). Therefore, the identification of new biomarkers and therapeutic approached is important.

A recent study revealed that the levels of HIFA-AS1 and XIST were upregulated in non-small cell lung cancer (NSCLC), and could be used as predictive biomarkers for NSCLC screening (Tantai et al. 2015). Sun et al. (2019) showed that TCF-4 targeting XIST was strongly correlated with lung cancer progression and macrophage polarization. Additionally, Wang et al. (2017b) showed that suppression of XIST inhibited cell growth and invasion, and induced apoptosis through reciprocal inhibition of miR-186-5p, which could be a new therapeutic biomarker in NSCLC. Many studies have since revealed the essential role of the lncRNA-miRNA-mRNA pathway in lung cancer. Specifically, it has been reported that XIST promoted lung cancer cell activities by upregulating BCL-2 (Fig. 2D), iASSP (Fig. 2E), LARP1 (Fig. 2F), Notch1 (Fig. 2G), RING1 (Fig. 2H), SOD2 (Fig. 2I), CDK8 (Fig. 2J), CBLL1 (Fig. 2K), MDM2 (Fig. 2L), and PAX6 (Fig. 2M) via regulation of miR-449a (Zhang et al. 2017b), miR-140 (Tang et al. 2017), miR-374a (Xu et al. 2017), miR-137 (Wang et al. 2018), miR-744 (Wang et al. 2019), miR-335 (Liu et al. 2019), miR-16 (Zhou et al. 2019), miR-212-3p (Qiu et al. 2019), miR-363-3p (Rong et al. 2020), and miR-142-5p (Jiang et al. 2020), respectively.

In addition, XIST influences lung cancer chemoresistance. Sun et al. (2017) demonstrated that XIST expression was upregulated in cisplatin-resistant cells. XIST promotes cell growth through BAG-1-mediated chemoresistance by antagonizing let-7i in lung adenocarcinoma (Fig. 2N). Another group confirmed that XIST regulated autophagy through miR-17/ATG7 signals in NSCLC. Downregulation of XIST overcomes chemoresistance by inhibition of autophagy in NSCLC (Fig. 2O) (Sun et al. 2017). In addition, Tian et al. (2019) showed that knockdown of XIST inhibited chemoresistance-associated cell growth and migration, as well as induced apoptosis in NSCLC. Upregulation of XIST is correlated with cisplatin resistance through the miR-144-3p/MDR1 signal pathway (Fig. 2P). Furthermore, Xu et al. (2020) concluded that suppression of XIST promoted cisplatin (DDP) chemosensitivity by combining with SMAD2 to inhibit NLRP3 and p53 transcription. Therefore, XIST might be used as a key biomarker to predict DDP efficacy in NSCLC. Recently, it was reported that XIST competitively bound to miR-520 in regulating DDP resistance through BAX, and was in turn involved in apoptosis via the p53 signaling pathway (Liu et al. 2021).

In summary, the data implied that XIST had a vital role in lung cancer progression and functioned as a diagnostic and therapeutic target for lung cancer.

### The role of XIST in nervous system tumors

#### XIST in glioma

Gliomas are the most common invasive nervous system tumors. Despite surgery combined with chemoradiotherapy, the survival of patients with gliomas is approximately 15 months (Thomas et al. 2014). Therefore, it is important to further investigate the gene regulatory networks to improve the treatment of gliomas.

Emerging evidence has shown that XIST expression was elevated in gliomas. Suppression of XIST inhibits tumor progression by reducing cell growth, invasion, and migration, as well as inducing apoptosis, which is mediated by miR-152 in gliomas (Yao et al. 2015). A subsequent study showed that XIST silencing suppressed glioma metastasis and angiogenesis in vivo and in vitro, which was mediated by miR-429 (Cheng et al. 2017).

**Fig. 3:**
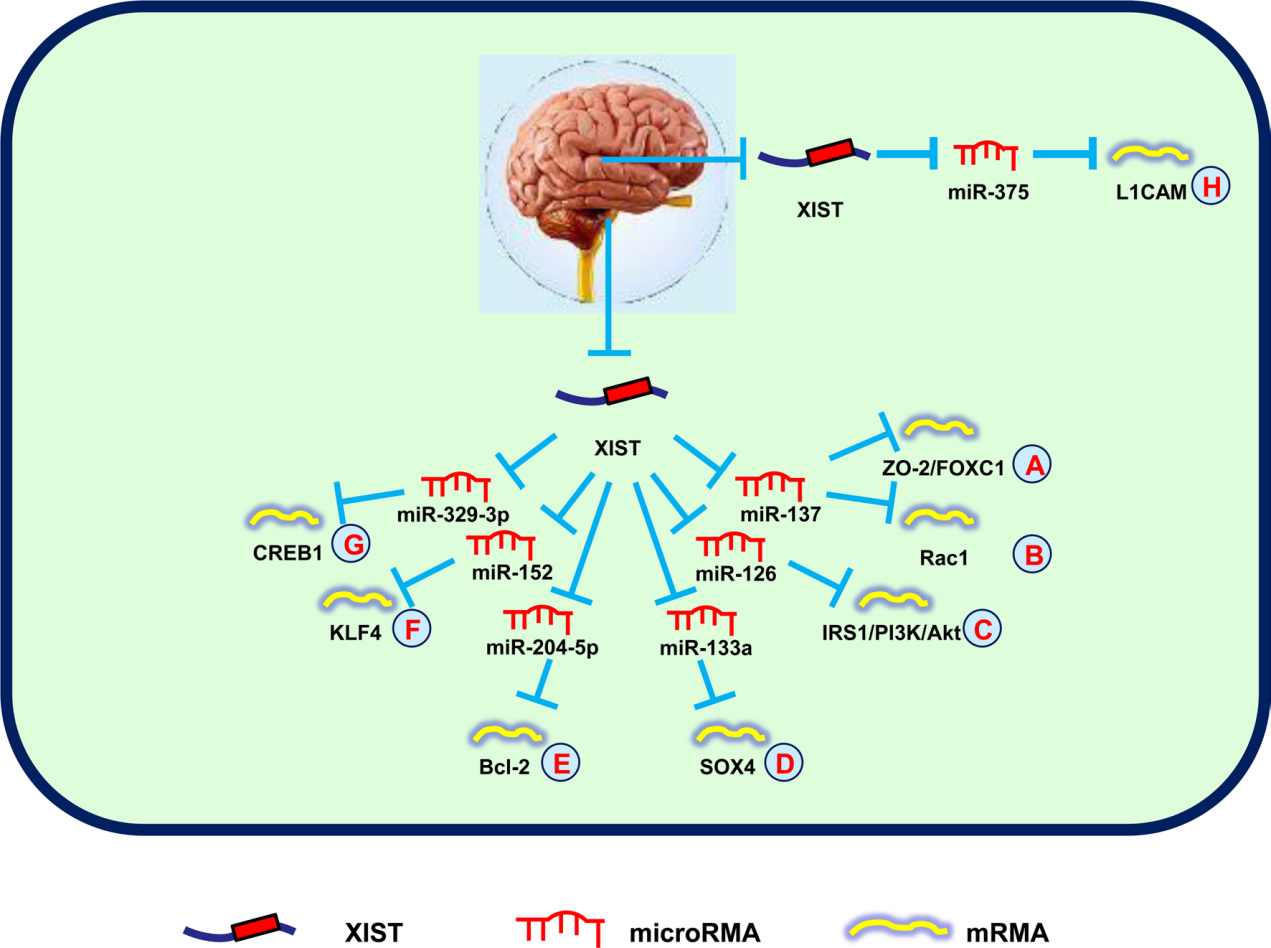
XIST mediates mechanisms involved in nervous system tumors.** A** XIST could promote the expression of ZO-2/FOXC1 by targeting miR-137. **B** XIST could promote the expression of Rac1 by targeting miR-137. **C** XIST could promote the expression of IRS1/PI3K/Akt by targeting miR-126. **D** XIST could promote the expression of SOX4 by targeting miR-133a. **E** XIST could promote the expression of Bcl-2 by targeting miR-204-5p. **F** XIST could promote the expression of KLF4 by targeting miR-152. **G** XIST could promote the expression of CREB1 by targeting miR-329-3p. **H** XIST could promote the expression of L1CAM by targeting miR-375

Recently, the lncRNA-miRNA-mRNA networks in gliomas have been elucidated. Yu et al. (2017) reported that downregulation of XIST limited cell angiogenesis by regulating FOXC1 and ZO-2 expression, which increased miR-137 expression in gliomas (Fig. 3A). Moreover, Rac1 is a target of the XIST-miR-137 regulatory axis, which is involved in glioma cell proliferation (Fig. 3B) (Wang et al. 2017c). Another study showed that XIST silencing suppressed cell viability, invasion, migration, and glucose metabolism in gliomas. Silencing of XIST decreases tumorigenicity through the lncRNA-XIST/miR-126/IRS1/PI3K/Akt axis in gliomas (Fig. 3C) (Cheng et al. 2020). Moreover, the XIST/miR-133a/SOX4 axis also promotes cell growth, metastasis, and the epithelial-mesenchymal transition (EMT), providing a novel target for glioma treatment (Fig. 3D) (Luo et al. 2020). Recently, it was reported that downregulation of XIST suppressed cell growth, invasion, and migration, and accelerated apoptosis of glioma cells. Mechanistic investigations showed that XIST, miR-204-5p, and Bcl-2 formed a network to regulate cell progression in gliomas (Fig. 3E) (Shen et al. 2020). Additionally, SRC-1 was shown to elevate glioblastoma stemness by modulating the XIST/miR-152/KLF4 axis, which provided a new diagnostic and therapeutic biomarker for glioblastomas (Fig. 3F) (Gong et al. 2021).

Chemotherapy and radiotherapy also have important roles in the treatment of gliomas. A recent study revealed that XIST enhanced glioma cell chemoresistance to temozolomide via regulating miR-29c/SP1/MGMT axis, which could be a novel target for glioma treatment (Du et al. 2017). Another research group reported that XIST expedited cell growth and invasion, and suppressed cell apoptosis through inhibiting the radiosensitivity of gliomas by increasing CREB1 expression via sponging miR-329-3p (Fig. 3G) (Wang et al. 2020c).

In summary, these data suggested that XIST played an important role in glioma progression, and functioned as a novel therapeutic target.

#### XIST in neuroblastoma (NB)

NBs consist of undifferentiated neuroblasts, while NBs from different sites have different clinical symptoms (Swift et al. 2018). NBs often occur in children < 5 years of age and is associated with cellular changes triggered by environmental stimuli; however, only 1–2% of NB patients have a family history, and the vast majority of NB patients are caused by unknown factors (Swift et al. 2018). Hence, an overall understanding of NB development can facilitate NB surveillance.

Zhang et al. (2019b) demonstrated that XIST decreased DKK1 expression through EZH2, thus accelerating the proliferation, migration, and invasion of NB cells. A subsequent study showed that XIST knockdown limited cell growth and elevated radiosensitivity of NBs by regulating the miR-375/L1CAM signals, thereby confirming that XIST may be a promising biomarker for NBs (Fig. 3H) (Yang et al. 2020).

Taken together, these results indicated that XIST had an oncogenic role in NBs.

### Oncogenic role of XIST in other systems

#### XIST in osteosarcoma (OS)

OSs are a common type of bone tumor in children and young adults that is derived from mesenchymal tissues of bone. OSs account for 15% of diagnosed malignancies in children and adolescents worldwide and severely affect their health (Aljubran et al. 2009). Epidemiological statistics have shown that when metastasis occurs, survival rates decrease from 65–70% to 19–30% (Aljubran et al. 2009). Therefore, identifying novel therapeutic targets and the underlying physiologic mechanism are important for OS treatment.

A study revealed that upregulation of XIST was associated with advanced clinical stage, advanced tumor size, distant metastasis, and poor overall survival rate (Li et al. 2017). Another study reported that XIST promoted cell growth by regulating P21 expression, thus serving as a potential biomarker of OSs (Xu et al. 2017). Similarly, inhibition of XIST suppresses cell growth, migration, and invasion in OSs (Wang et al. 2019). Moreover, knockdown of XIST induces cell apoptosis via modulation of the NF-kB/PUMA axis in OSs (Gao et al. 2019). Li et al. (2019b) reported that XIST promotes cell growth and invasion via suppression of miR-137 expression, thus providing a potential therapeutic target for OSs.

**Fig. 4:**
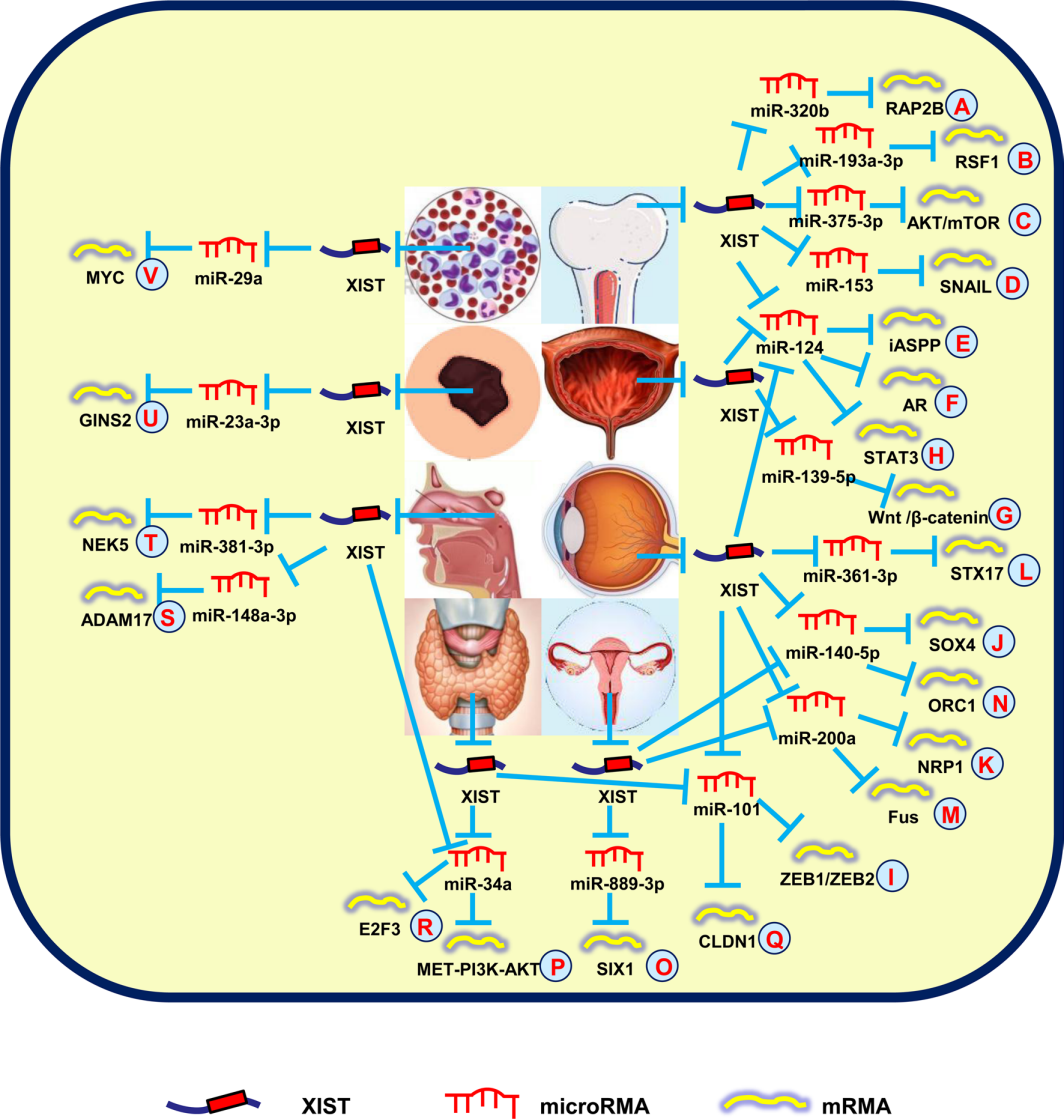
XIST mediates mechanisms involved in other system tumors. **A** XIST could promote the expression of RAP2B by targeting miR-320b. **B** XIST could promote the expression of RSF1 by targeting miR-193a-3p. **C** XIST could promote the expression of AKT/mTOR by targeting miR-375-3p. **D** XIST could promote the expression of SNAIL by targeting miR-153. **E** XIST could promote the expression of iASPP by targeting miR-124-3p. **F** XIST could promote the expression of AR by targeting miR-124. **G** XIST could promote the expression of Wnt/β-catenin by targeting miR-139-5p. **H** XIST could promote the expression of STAT3 by targeting miR-124. **I** XIST could promote the expression of ZEB1/ZEB2 by targeting miR-101. **J** XIST could promote the expression of SOX4 by targeting miR-140-5p. **K** XIST could promote the expression of NRP1 by targeting miR-200a-3p. **L** XIST could promote the expression of STX17 by targeting miR-361-3p. **M** XIST could promote the expression of Fus by targeting miR-200a. **N** XIST could promote the expression of ORC1 by targeting miR-140-5p. **O** XIST could promote the expression of SIX1 by targeting miR-889-3p. **P** XIST could promote the expression of MET-PI3K-AKT by targeting miR-34a. **Q** XIST could promote the expression of CLDN1 by targeting miR-101-3p. **R** XIST could promote the expression of E2F3 by targeting miR-34a-5p. **S** XIST could promote the expression of ADAM17 by targeting miR-148a-3p. **T** XIST could promote the expression of NEK5 by targeting miR-381-3p. **U** XIST could promote the expression of GINS2 by targeting miR-23a-3p. **V** XIST could promote the expression of MYC by targeting miR-29a

Recently, a series of studies showed that XIST promoted OS cell development by the lncRNA-miRNA-mRNA networks. Lv et al. (2018) first reported that XIST was responsible for cell growth and invasion by modulating the miR-320b/RAP2B axis in OSs (Fig. 4A). Another group revealed that XIST functioned as a ceRNA to sequester miR-193a-3p expression, which modulated the target gene, RSF1 (Fig. 4B) (Wu et al. 2017b). In addition, silencing of XIST suppresses cell proliferation and autophagy by inhibiting the AKT/mTOR axis and antagonizing miR-375-3p in OSs (Fig. 4C) (Sun et al. 2019). Shortly thereafter, XIST was shown to promote cell migration, invasion, and EMT by regulating miR-153/SNAIL signaling, thus serving as a novel therapeutic biomarker for OSs (Fig. 4D) (Wen et al. 2020). Recently, Hai et al. (2021) reported that overexpressed XIST promoted iASPP expression to stimulate cell proliferation by sponging miR-124-3p in OSs (Fig. 4E).

These findings showed that XIST is a promising therapeutic target for inhibiting OS progression.

#### XIST in bladder cancer (BC)

BCs are the 10th most common tumors worldwide, with an estimated 573,278 new cases and 212,536 deaths in 2021 (Siegel et al. 2021). BCs are approximately four times more common in men than in women. Although cigarette smoking, exposure to chemical products, chronic infections, aromatic amines, and other pathogenic factors have been identified, the specific pathologic mechanism underlying BC development is unknown (Siegel et al. 2021). Thus, it is essential to identify new diagnostic biomarkers and the molecular mechanism underlying BCs.

Xu et al. (2018a) showed that silencing XIST suppressed cell clone formation and EMT in BCs, which was partially restored by miR-200c knockdown. Another group reported that XIST knockdown reduced p53 expression to inhibit cell growth and migration through combination with TET1 in BCs (Hu et al. 2019). Moreover, XIST promotes cell migration and growth by antagonizing miR-133a, suggesting a possible therapeutic marker for BCs (Zhou et al. 2019). The XIST-miRNA-mRNA regulatory networks have also been shown to be present in BCs. Xiong et al. (2017) indicated that XIST stimulated cell proliferation, migration, and invasion through miR-124-dependent androgen receptor modulation (Fig. 4F). Another group reported that downregulation of XIST impaired cell proliferation and metastasis by modulating the miR-139-5p-mediated Wnt/β-catenin axis in BCs (Fig. 4G) (Hu et al. 2017). Recently, targeting the XIST/miR-335 axis was shown to elevate the anti-tumor effects of platycodin D in BCs, thus suggesting a therapeutic strategy for BCs (Chen et al. 2020).

Overall, the data indicated that XIST acts as an oncogene in BC progression.

#### XIST in retinoblastoma (RB)

RBs are the most common intraocular malignancies in infants and young children. RBs are derived from photoreceptor precursor cells and are prone to intracranial and distant metastasis. Patients with early RBs can undergo surgical treatment, or receive radiotherapy, chemotherapy, or other comprehensive measures; however, advanced RBs often lead to a poor prognosis. Therefore, it is necessary to identify effective diagnostic targets and treatment strategies for RBs.

Lyu et al. (2019) reported that lncRNA NKILA might suppress the growth, invasion, and migration of RBs via XIST knockdown. Upregulation of XIST accelerates cell growth, invasion, and EMT by modulating miR-142-5p expression (Xu and Tian 2020).

Recently, a large number of studies indicated that XIST stimulated RB cell progression via the lncRNA-miRNA-mRNA axis. Hu et al. (2018) reported that XIST expedited cell growth by regulating the miR-124/STAT3 axis in RBs (Fig. 4H). In addition, XIST elevates the development of RB by functioning as a miR-101 sponge to regulate ZEB1/ZEB2 expression and providing a new therapeutic choice for RBs (Fig. 4I) (Cheng et al. 2019). Another study pointed out that XIST was upregulated in RBs, and suppression of XIST inhibited cell growth and invasion through miR-140-5p/SOX4 signals, suggesting a novel understanding of the mechanism underlying RB (Fig. 4J) (Wang et al. 2020d). Also, XIST silencing increases the PI3K-Akt/MAPK-ERK axis mediated by miR-200a-3p, which affects cell growth, invasion, apoptosis, and EMT in RBs (Fig. 4K) (Zhao et al. 2020). Subsequent studies showed that XIST enhanced the aggressive phenotype of RB cells through miR-361-3p/STX17 signals, functioning as an oncogenic lncRNA (Fig. 4L) (Yang et al. 2020).

XIST is also involved in the chemosensitivity impact on RB patients. Yao et al. (2020) showed that XIST expression was increased in RB tissues. Silencing of XIST inhibits cell development and expedites vincristine sensitivity via functioning as a miR-204-5p sponge, highlighting a potential therapeutic target for RBs.

These findings verified that XIST participates in RB development and functions as a therapeutic and prognostic biomarker.

#### XIST in cervical cancer (CC)

CC is the 4th most frequently diagnosed tumor and the 6th leading cause of cancer death in women, with an estimated 66,570 new cases and 12,940 deaths in the United States in 2021 (Turner et al. 2020). Although tumor screening and HPV vaccination have reduced the incidence of CC, the pathogenesis and treatment warrant further study.

Zhu et al. (2018a) reported that XIST promoted CC progression by regulating the miR-200a/Fus axis while serving as a ceRNA (Fig. 4M). Another group showed that downregulation of XIST inhibited cell growth through miR-140-5p/ORC1 signals in CC (Fig. 4N) (Chen et al. 2019b). Additionally, XIST silencing also impairs cell growth, invasion, and migration by modulating the miR-889-3p/SIX1 signaling pathway and serving as a novel target for the progression of new countermeasures of CC (Fig. 4O) (Liu et al. 2020).

The underlying mechanism of XIST in CC still needs further study.

#### XIST in thyroid cancer

The incidence of thyroid cancer is 586,202 cases worldwide, ranking 9th in 2021 (Siegel et al. 2021). Women are three times more likely to have thyroid cancer than men, but the death rate is not high (Siegel et al. 2021). The only identified risk factor for thyroid cancer is ionizing radiation. Therefore, improving thyroid cancer treatment, including medications and surgery, is a pressing problem.

Xu et al. (2018b) showed that abnormally overexpressed XIST was positively correlated with lymph node metastasis and TNM stage. The molecular mechanism indicated that XIST functions as an oncogene with respect to growth, invasion, and migration by modulating miR-141 in thyroid cancer. A few months later, another research group reported that XIST functioned as a miR-34a sponge, competing with MET to regulate cell growth in thyroid cancer (Fig. 4P) (Liu et al. 2018). Furthermore, XIST facilitates cell invasion and migration by directly modulating the miR-101-3p/CLDN1 axis and providing new insight into thyroid cancer treatment (Fig. 4Q) (Du et al. 2021).

Overall, these data indicated that XIST has an oncogenic role in thyroid cancer and represents a new therapeutic target.

#### XIST in nasopharyngeal carcinoma (NPC)

NPC is the most common malignant tumor of the oral cavity and pharynx, and squamous cell carcinoma is the most common pathological type. Early stage NPC is easy to be misdiagnosed because of its hidden location and lack of features. Therefore, it is essential to identify an diagnostic marker for early-stage NPC.

A recent study revealed that XIST was upregulated in NPC and led to shorter survival time. An analysis of the mechanism NPC development indicated that XIST promoted NPC cell growth in part by increasing E2F3 through antagonizing miR-34a-5p expression (Fig. 4R) (Song et al. 2016). Another study pointed out that suppression of XIST inhibited cell growth and increased radiosensitivity of NPC cells by upregulating miR-29c expression, offering a novel therapeutic strategy for NPC patients (Han et al. 2017). Cheng et al. (2018) reported that downregulation of XIST induced cell apoptosis and suppressed cell growth and invasion by sponging miR-491-5p in NPC. In addition, Shi et al. (Shi et al. 2020) demonstrated that knockdown of XIST suppressed cell proliferation, metastasis, and EMT by regulating the miR-148a-3p/ADAM17 signal pathway in NPC (Fig. 4S). Moreover, Zhao et al. (2020) indicated that downregulation of XIST inhibited cell glycolysis, migration, and invasion by modulating the miR-381-3p/NEK5 axis in NPC (Fig. 4T).

These data indicate that XIST might be used as a prognostic biomarker and therapeutic target for NPC.

#### XIST in melanoma

The estimated incidence of melanomas in 2021 is the 5th highest among malignancies in both men and women (Siegel et al. 2021). Excessive ultraviolet exposure is thought to be the most important reason for the sharp rise in melanoma rates, which accounts for approximately 90% of skin cancer deaths. Therefore, it is important to explore early diagnostic and therapeutic biomarkers for melanomas.

Hao et al. (2021) revealed that XIST facilitated oncogenic behavior of melanomas by sequestering miR-23a-3p and indirectly targeting its downstream gene, GINS2, thus providing a potential target (Fig. 4U).

This result shows that XIST serves as an oncogene to accelerate melanoma progression.

#### XIST in leukemia

Leukemia has the sixth (male) and eighth (female) highest mortality rate among malignancies, but morbidity ranks 9th (male) and 10th (female). The causes of leukemia are not fully understood. The mutations of some genes in hematopoietic cells caused by various causes lead to the formation of clonal abnormal hematopoietic cells. Therefore, it is imperative to identify therapeutic targets for leukemia.

Wang et al. (2020a) reported that XIST was increased in acute myeloid leukemia (AML) cells. Functional assays demonstrated that XIST knockdown decreased the expression of MYC by antagonizing miR-29a, thereby suppressing viability, reducing chemoresistance, and promoting apoptosis of AML cells (Fig. 4V).

In summary, this study offers new insights into the mechanism by which XIST regulates leukemia.

## Conclusion and future perspectives

Cancer refers to a tumor formed by abnormal proliferation of cells in the local tissue under the stimulation of various tumorigenic factors. Malignant tumors can destroy the structure and function of tissues and organs, causing necrosis and hemorrhage combined with infection, and the patient may eventually die due to organ failure. The Union for International Cancer Control has put forward the concept of tertiary prevention of malignant tumors. The secondary prevention of tumors is how to detect cancer at an early stage and treat it promptly. Therefore, many researchers have carried out studies on early diagnostic biomarkers and therapeutic targets of tumors.

LncRNAs have been demonstrated to be abnormally expressed in many diseases, including cancers, functioning as oncogenes or tumor suppressors. Recently, it has been repeatedly shown that XIST was overexpressed in various tumors, acting as an oncogenic lncRNA (Wu et al. 2017a; Chen et al. 2016; Zhang et al. 2019a). The overexpression of XIST is related to the onset and development of tumors, such as cell viability, autophagy, clone formation, angiogenesis, proliferation, migration, invasion, apoptosis, EMT, metastasis, tumor glycolysis, cell cycle regulation, radiosensitivity, chemoresistance, and stem cell formation. In contrast, XIST knockdown significantly inhibits the aggressive phenotypes of those tumors. Therefore, XIST is considered to be a potential biomarker and therapeutic target of tumors; however, the mechanistic investigations of XIST need to be further explored.

In our review, we summarized the carcinogenesis of XIST in multiple tumors. First, XIST promotes tumor development and prognosis by interacting with miRNAs and/or targeting proteins. The overexpression of XIST is closely related to the clinicopathologic features of tumors. Second, XIST competitively binds to mRNAs by adsorbing miRNAs, which constitutes a regulatory model of the “XIST-miRNAs-mRNAs” axis. Finally, XIST plays an important role in the sensitivity of tumor radiotherapy and chemotherapy, which can be targeted to improve the efficacy of tumor radiotherapy and chemotherapy.

It should be noticed that there existed some limitations in our manuscript. First, XIST was overexpressed in many non-sex-related human cancers and downregulated in some sex-specific cancers, such as breast cancer, ovarian cancer, and prostate cancer (Zhang et al. 2020; Guo et al. 2021; Du et al. 2017). Whether this phenomenon is related to the fact that XIST is a key regulator of mammalian XCI deserves further study. Another reason may be due to the differences in the source of tumor tissue and extracellular microenvironment. Second, in the same type of tumor, such as HCC, OS, and renal cell carcinoma, the expression of XIST was also inconsistent (Lin et al. 2018; Zhang and Xia 2017; Sun et al. 2019). These inconsistencies could be due to sex differences between samples, or XIST controls cancer development at multiple levels. Finally, there were not enough data to fully confirm the relationship between XIST and clinicopathological features. Therefore, larger and multicenter studies are needed to determine this correlation.

In all, our review demonstrated that XIST is overexpressed in most human tumors and involved in multiple layers of carcinogenesis. The effects of XIST on tumors are complex, including many tumorigenic regulatory networks and tumor-associated miRNAs. Therefore, a sufficient understanding of XIST in molecular biology will be helpful for the utilization of XIST as a diagnostic biomarker and therapeutic target for clinical cancer therapy.

## Data Availability

Not applicable.

## Abbreviations

LncRNA: Long non-coding RNA
XIST: X-inactive specific transcript
XCI: X-chromosome inactivation
EC: Esophageal cancer
GC: Gastric cancer
CRC: Colorectal cancer
HCC: Hepatocellular carcinoma
NB: Neuroblastoma
OS: Osteosarcoma
BC: Bladder cancer
RB: Retinoblastoma
CC: Cervical cancer
NPC: Nasopharyngeal carcinoma
ESCC: Esophageal squamous cell carcinoma
5FU: 5-Fluorouracil
LSCC: Laryngeal squamous cell carcinoma
NSCLC: Non-small cell lung cancer
DDP: Cisplatin
EMT: Epithelial-mesenchymal transition
AML: Acute myeloid leukemia
